# Extensive pipeline location data resource: Integrating reported incidents, past environmental loadings, and potential geohazards for integrity evaluations in the U.S. Gulf of Mexico

**DOI:** 10.1016/j.dib.2024.110728

**Published:** 2024-07-09

**Authors:** Isabelle Pfander, Lucy Romeo, Rodrigo Duran, Alec Dyer, Catherine Schooley, Madison Wenzlick, Patrick Wingo, Dakota Zaengle, Jennifer Bauer

**Affiliations:** a National Energy Technology Laboratory, 1450 Queen Avenue SW, Albany, OR 97321, USA; b NETL Support Contractor, 1450 Queen Avenue SW, Albany, OR 97321, USA; c Theiss Research, 7411 Eads Avenue, La Jolla, CA 92037, USA

**Keywords:** Infrastructure, Offshore, Risk prevention, Oil and gas, Oceanography, Extreme weather, Submarine landslides, Machine learning

## Abstract

The U.S. Gulf of Mexico contains a complex network of existing, decommissioned, and abandoned oil and gas pipelines, which are susceptible to a number of stressors in the natural-engineered offshore system including corrosion, environmental hazards, and human error. The age of these structures, coupled with extreme weather events increasing in intensity and occurrence from climate change, have resulted in detrimental environmental and operational impacts such as hydrocarbon release events and pipeline damage. To support the evaluation of pipeline infrastructure integrity for reusability, remediation, and risk prevention, the U.S. Gulf of Mexico Pipeline and Reported Incident Datasets were developed and published. These datasets, in addition to supporting advanced analytics, were constructed to inform regulatory, industry, and research stakeholders. They encompass more than 490 attributes relating to structural information, incident reports, environmental loading statistics, seafloor factors, and potential geohazards, all of which have been spatially, and in some cases temporally matched to more than 89,000 oil and gas pipeline locations. Attributes were acquired or derived from publicly available, credible resources, and were processed using a combination of manual efforts and customized scripts, including big data processing using supercomputing resources. The resulting datasets comprise a spatial geodatabase, tabular files, and metadata. These datasets are publicly available through the Energy Data eXchange®, a curated online data and research library and laboratory developed by the U.S. Department of Energy's National Energy Technology Laboratory. This article describes the contents of the datasets, details the methods involved in processing and curation, and suggests application of the data to inform and mitigate risk associated with offshore pipeline infrastructure in the Gulf of Mexico.

Specifications Table

**Table:** 

Subject	Energy, Energy Engineering and Power Technology, Safety, Risk, Reliability and Quality
Specific subject area	Energy infrastructure, specifically oil and gas pipelines in the U.S. Gulf of Mexico.
Data format	Raw, Analyzed, Filtered, Geospatially processed
Type of data	Table, Graph, Figure, Geospatial data
Data collection	Data were acquired from credible, publicly available online resources through manual access as well as customized Python scripts for larger datasets (e.g., environmental loadings). In addition, biochemical data from the Model of Ecosystem Dynamics, Nutrient Utilization, Sequestration and Acidification 2.0 model were obtained directly from the United Kingdomʼs National Oceanography Center, transferring the data through a private Energy Data eXchange® (EDX) workspace using the EDX API. All data collection efforts targeted the U.S. Gulf of Mexico.
Data source location	Primary data sources include: U.S. Department of Energy, National Energy Technology Laboratory, Pipeline and Hazardous Materials Safety Administration, Bureau of Safety and Environmental Enforcement, National Oceanic and Atmospheric Administration, Hybrid Coordinate Ocean Model, International Best Track Archive for Climate Stewardship, Model of Ecosystem Dynamics, nutrient Utilization, Sequestration and Acidification, National Center for Atmospheric Research European Center for Medium-Range Weather Forecasts, Institute of Arctic and Alpine Research, and Central and Eastern United States Seismic Source Characterization for Nuclear Facilities. Full citations can be found in the published metadata. Lucy Romeo, Isabelle Pfander, Rodrigo Duran, Michael Sabbatino, Catherine Schooley, Madison Wenzlick, Patrick Wingo, Dakota Zaengle, Jennifer Bauer, U.S. Gulf of Mexico Pipeline and Reported Incident Datasets, 1/11/2024, https://edx.netl.doe.gov/dataset/u-s-gulf-of-mexico-pipeline-and-reported-incident-datasets, DOI: 10.18141/2280823
Data accessibility	Repository name: Energy Data eXchange® (EDX) Data identification number: DOI: 10.18141/2280823 Direct URL to data: https://edx.netl.doe.gov/dataset/u-s-gulf-of-mexico-pipeline-and-reported-incident-datasets Instructions for accessing these data: This resource is publicly available on EDX and is licensed as Creative Commons. The spatial database, tabular datasets, documentation, and data dictionaries can be downloaded individually or as a compressed file.
Related research article	

## 1 Value of the Data

• The U.S. Gulf of Mexico Pipeline and Reported Incident Datasets is a comprehensive, publicly available resource containing spatial and tabular data that represent offshore existing, abandoned, or removed oil and gas pipelines. The datasets include reported incidents compiled from regulatory agencies, and were developed to support government, regulatory, commercial, and public stakeholders.
• The datasets’ design enables an interoperable approach, future research, and risk assessments. The datasets represent potential infrastructure stressors spanning the offshore natural-engineered system of the Gulf of Mexico where oil and gas pipeline locations are spatially and temporally joined with information related to structural characteristics, reported incidents, environmental loadings, seafloor factors, and geohazards.
• Datasets were designed to inform the National Energy Technology Laboratory's Advanced Infrastructure Integrity Modelling tool, which employs big data computing and multiple machine learning models to evaluate the integrity, remediation needs, and potential risk of energy infrastructure.
• The pipeline incident dataset is a compilation of reported pipeline-related incidents in a standardized resource that can be applied to assess leading risk factors for offshore infrastructure incidents, including costly ruptures or release events. Moreover, this resource may be used to inform regulatory and commercial entities where failures are more likely to occur, supporting the prioritization of abandonment, removal, or repurposing of oil and gas pipelines.
• The datasets were designed to inform infrastructure reusability and repurposing assessment to support alternative energy strategies, mitigating emissions, and slowing climate change. These datasets are particularly relevant considering the ongoing energy transition and growing carbon capture and storage and hydrogen economy.

## 2 Background

A 2021 report by the United States (U.S.) Government Accountability Office noted a need for an up-to-date and reliable federal oversight of oil and gas pipeline integrity in the U.S. Gulf of Mexico (GoM) due to previous regulations resulting in more than 18,000 miles of abandoned pipelines remaining on the seafloor [1]. Moreover, as the GoM is a productive energy hub, existing infrastructure are of interest to support the growing carbon, hydrogen, and alternative energy economies. This includes carbon capture and storage (CCS) potential, where existing energy infrastructure might offer cost-effective reuse and repurposing opportunities [2].

Amidst the federal oversight needs and energy transitions, the U.S. Department of Energy's (DOE) National Energy Technology Laboratory (NETL) has published the U.S. Gulf of Mexico Pipeline and Reported Incident Datasets to support the evaluation of infrastructure integrity [3]. These datasets include potential pipeline stressor information, such as factors related to corrosion, environmental loadings, past incidents, and landslide likelihood, due to their influence in damaged or destroyed pipeline infrastructure and resulting economic and environmental impacts [4]. Designed to inform reuse opportunities, remediation needs, and risk prevention, these datasets provide an integrated, publicly available resource to assess the state of the region's pipeline networks.

## 3 Data Description

### 3.1 Datasets structure

**Fig. 1:**
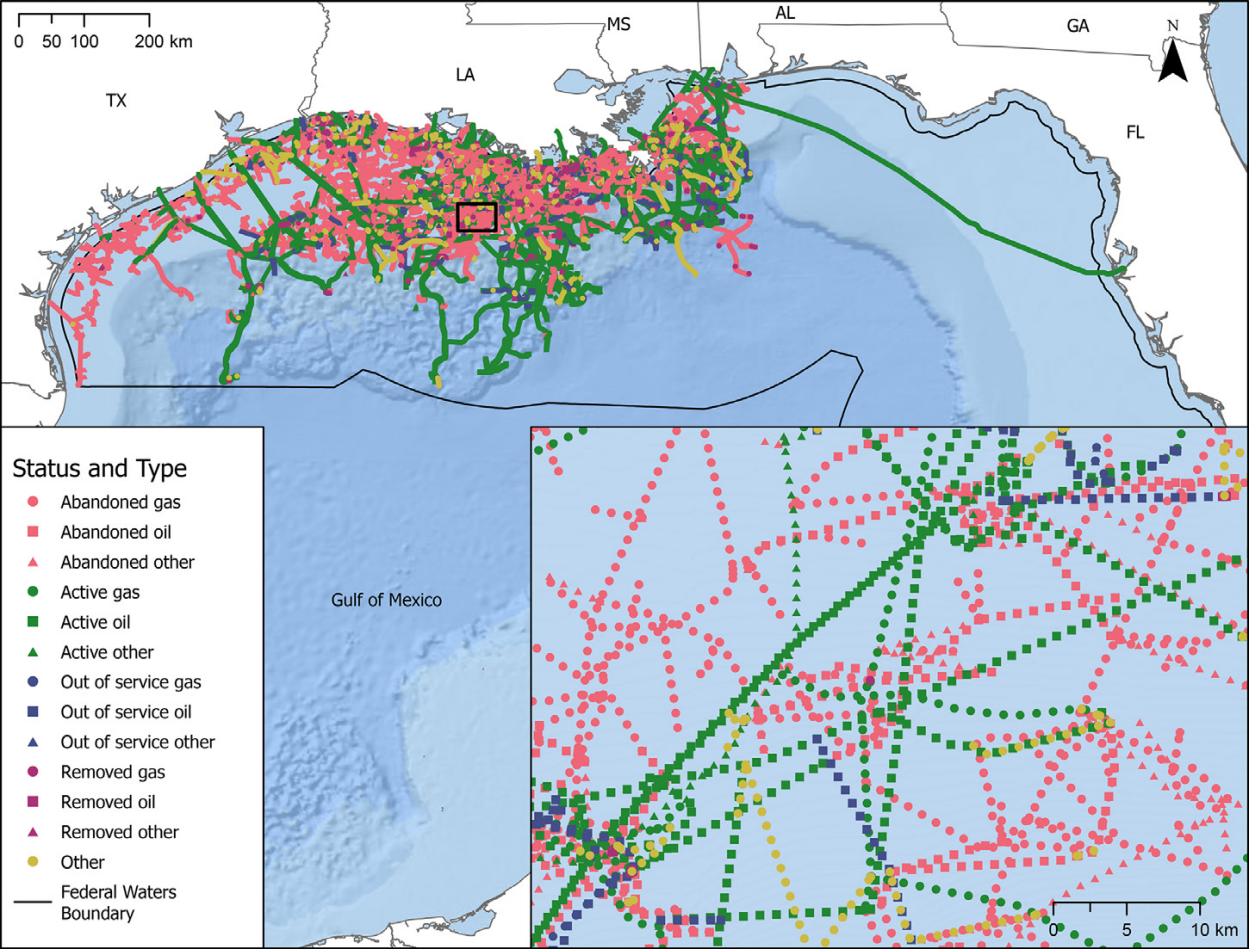
Oil and gas pipeline point locations in the U.S. GoM, symbolized by type (color) and status (shape). A zoomed-in map is provided to better visualize the pipeline point locations, where each point represents a pipeline location per kilometer along the pipeline, including end points.

**Fig. 2:**
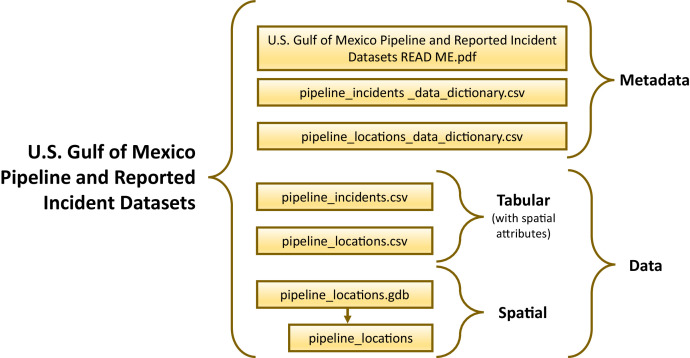
Framework of the published and publicly available U.S. Gulf of Mexico Pipeline and Reported Incident Datasets.

The U.S. Gulf of Mexico Pipeline and Reported Incident Datasets provide spatial and tabular representations of the region's pipelines spanning more than 64,000 km (41,000 mi) across federal and state waters. The datasets include pipeline locations which have been installed by the oil and gas industry since the 1940s (Fig. 1), as well as a tabular dataset representing more than three decades of reported pipeline-related incidents. Resulting datasets, as shown in Fig. 2, were formatted to support future use and interoperability. Spatial data of pipeline point locations are formatted as a feature class (*pipeline_locations)* in a file geodatabase (*pipeline_locations.gdb*), and tabular data are formatted as Comma Separated Value (CSV) files, which represent pipeline point location records (*pipeline_locations.csv*) and standardized incident reports (*pipeline_incidents.csv*). Tabular field dictionaries are also included to describe fields and original source information for pipeline location records (*pipeline_location_data_dictionary.csv*), and to describe fields for pipeline incidents (*pipeline_incidents_data_dictionary.csv*). Resulting datasets, as shown in Fig. 2, were formatted to support future use and interoperability. A Portable Document Format (PDF) metadata file is available alongside the datasets as an overview file.

The point locations datasets contain 494 attributes (i.e., fields) representing various structural characteristics, environmental loading statistics, incident histories, and other variables representing potential impacts on structural stress. These attributes are tied to the 89,986 records (i.e., points) representing locations at every kilometer along existing and previously existing oil and gas pipelines, as well as pipeline end points. The spatially explicit representation, as illustrated in Fig. 1, enables greater spatial accuracy for integration and analysis, as opposed to summarizing features along the entire length of a pipeline. Data representing various potential pipeline stressors were spatially, and in some instances temporally, matched to the pipeline point locations. The associated data layers representing structural factors, reported incidents, environmental loadings, seafloor factors, and geohazards are discussed in the following sections (Fig. 2). In addition, the integrated incidents dataset provides supplemental information, where each record provides detail for one of the 943 pipeline-related reported incidents.

### 3.2 Pipeline data

Pipeline data were acquired through the U.S. Department of Interior's (DOI) Bureau of Safety and Environmental Enforcement's (BSEE) Data Center website (https://www.data.bsee.gov/). These data included a spatial polyline feature layer representing pipelines, as well as tabular resources providing pipeline structural characteristics. Acquired data were processed into point representations along existing and prior pipeline locations in the GoM (Fig. 1). The point representations enable spatially explicit analytics and evaluations along the available pipeline data. The spatial version of this dataset is referenced in the World Geodetic System (WGS) 1984 geographic coordinate system, based on the latitude (*POINT_Y*) and longitude (*POINT_X*) fields (Fig. 1). To support reusability, the dataset is also offered as a CSV file.

The pipeline point locations dataset provides spatially matched variables representing reported incidents, environmental loadings, and seafloor factors and potential geohazards (Fig. 2). Temporal matching was applied in instances where data was available. More information on these resources, available at each of the more than 89,000 points, are provided in the following sections.

### 3.3 Reported incidents

Regulatory agencies maintain records of past incidents involving pipeline infrastructure. Overseeing the transportation of hazardous materials through pipelines, the U.S. Department of Transportation Pipeline and Hazardous Materials Safety Administration (PHMSA) collects and maintains incident reports from operators to satisfy safety regulations. These incident reports are publicly available to download online (https://www.phmsa.dot.gov/data-and-statistics/pipeline/pipeline-incident-flagged-files). Similarly, BSEE regulates offshore energy production on the Outer Continental Shelf (OCS). Incidents reported to BSEE by operators are compiled into Investigation Reports and made publicly accessible online on their Data Center.

**Fig. 3:**
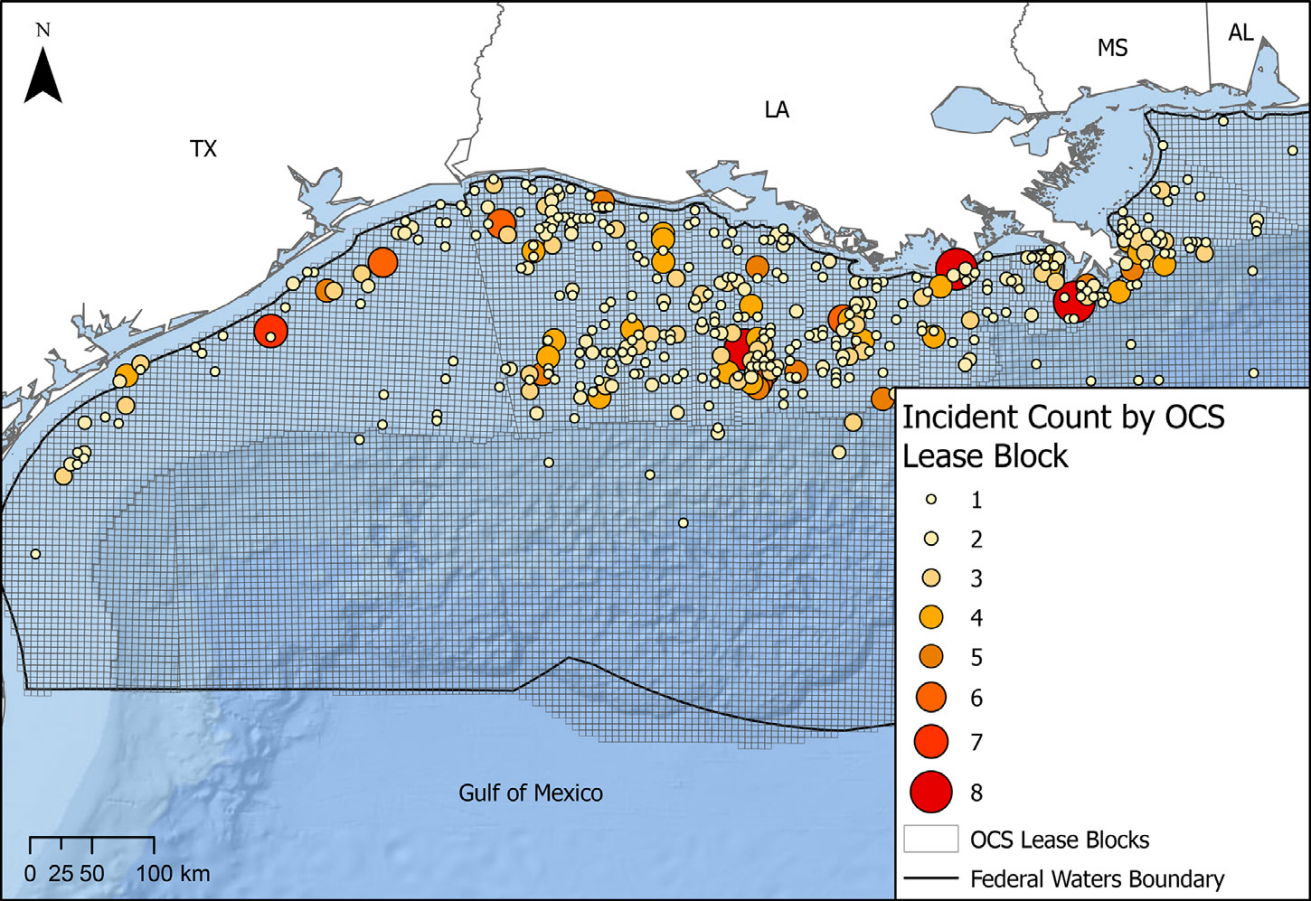
Map of GoM depicting the number of reported incidents per lease block from 1986 to 2021, created using data from the U.S. Gulf of Mexico Pipeline and Reported Incident Datasets.

Reported incidents obtained from PHMSA and BSEE were processed, merged, standardized, and summarized for publishing in the pipeline incident dataset. This resulted in a CSV file containing 85 fields representing more than 900 reported pipeline and pipeline-related incidents from 1986 to 2021. Fields represent the reported incident date, geography (e.g., OCS lease block as shown in Fig. 3), and directly reported information from operators (e.g., cost, fatalities or injuries, maximum operating pressure), as well as standardized and summarized attributes calculated by NETL (e.g., remediation presence, total cost converted to 2021 U.S. dollars (USD), preliminary severity scores).

The dataset includes NETL-standardized type and flag fields (e.g., presence or absence) of various potential causes and consequences of the incidents, including material failure, extreme weather, equipment cause, and corrective action based on reports and contextual notes. Built upon the derived cause and consequence fields, attributes representing incident severity serve as relative markers of the intensity of the incident related to the possible outcomes and level of mitigation needed. The preliminary severity scores were assigned using domain expertise of the pipeline hazards. The scores can be used in analyses to examine the pipeline and incident attributes and their potential effects on incident occurrence and level of severity. There are four severity measurements calculated from varying criteria based on 1) potential cause or consequence, 2) potential consequence, 3) reported total release amount, and 4) reported total cost (USD). Table 1 provides the NETL-standardized fields compared to the median results of the four severity scores across all incidents.

**Table 1:** Statistical summary of the potential causes and consequence fields, standardized across incident reports. Also included are the medians values of the four preliminary severity scores by reported potential cause or consequence.

				Median Severity Score			
	Field	Incident Count	Percent of Total Incidents	Based on Reported Potential Causes and Consequences	Based on Reported Potential Consequences	Based on Reported Damage Costs	Based on Reported Release Amount
Reported Potential Causes	Material Failure	506	53.66 %	0.26	0.22	0.60	0.00
	Internal Corrosion	355	37.65 %	0.26	0.22	0.60	0.00
	External Corrosion	94	9.97 %	0.30	0.26	0.60	0.20
	Incorrect Operation	195	20.68 %	0.30	0.22	0.60	0.00
	Extreme Weather	176	18.66 %	0.37	0.30	0.80	0.00
	Hurricane	164	17.39 %	0.37	0.35	0.80	0.00
	EquipmentCause	93	9.86 %	0.30	0.26	0.60	0.20
	Geohazard	48	5.09 %	0.33	0.24	0.60	0.00
	Fire	29	3.08 %	0.37	0.35	0.60	0.00
	Explosion	7	0.74 %	0.44	0.35	0.40	0.00
Reported Potential Consequences	Structural Damage	912	96.71 %	0.30	0.22	0.60	0.00
	Corrective Action	570	60.45 %	0.30	0.26	0.60	0.00
	Water Contamination	448	47.51 %	0.37	0.33	0.60	0.40
	Spill	393	41.68 %	0.37	0.35	0.60	0.40
	Pipeline Shutdown	384	40.72 %	0.37	0.35	0.60	0.40
	Platform Damaged	128	13.57 %	0.41	0.35	0.60	0.00
	Pipeline Replaced	93	9.86 %	0.33	0.30	0.60	0.00
	Pipeline Abandonment	63	6.68 %	0.59	0.57	0.60	0.00
	Pipeline Destroyed	53	5.62 %	0.59	0.52	0.60	0.00
	Platform Destroyed	31	3.29 %	0.63	0.65	0.80	0.00
	Remediation	22	2.33 %	0.35	0.30	0.60	0.40
	Wildlife Impact	3	0.32 %	0.37	0.35	0.60	0.60

In addition to the severity scores, incident data are matched to NETL-derived OCS lease blocks and state waters, and denote the reported lease block, latitude and longitude, and the written descriptions of the incidents. The standardized incident dataset (*pipeline_incidents.csv*) includes all incident fields and OCS lease blocks which allow for spatial analysis and matching to the pipeline segments. Fig. 3 visualizes the number of incidents in the GoM by OCS lease block, depicting the spatial distribution of the dataset.

**Fig. 4:**
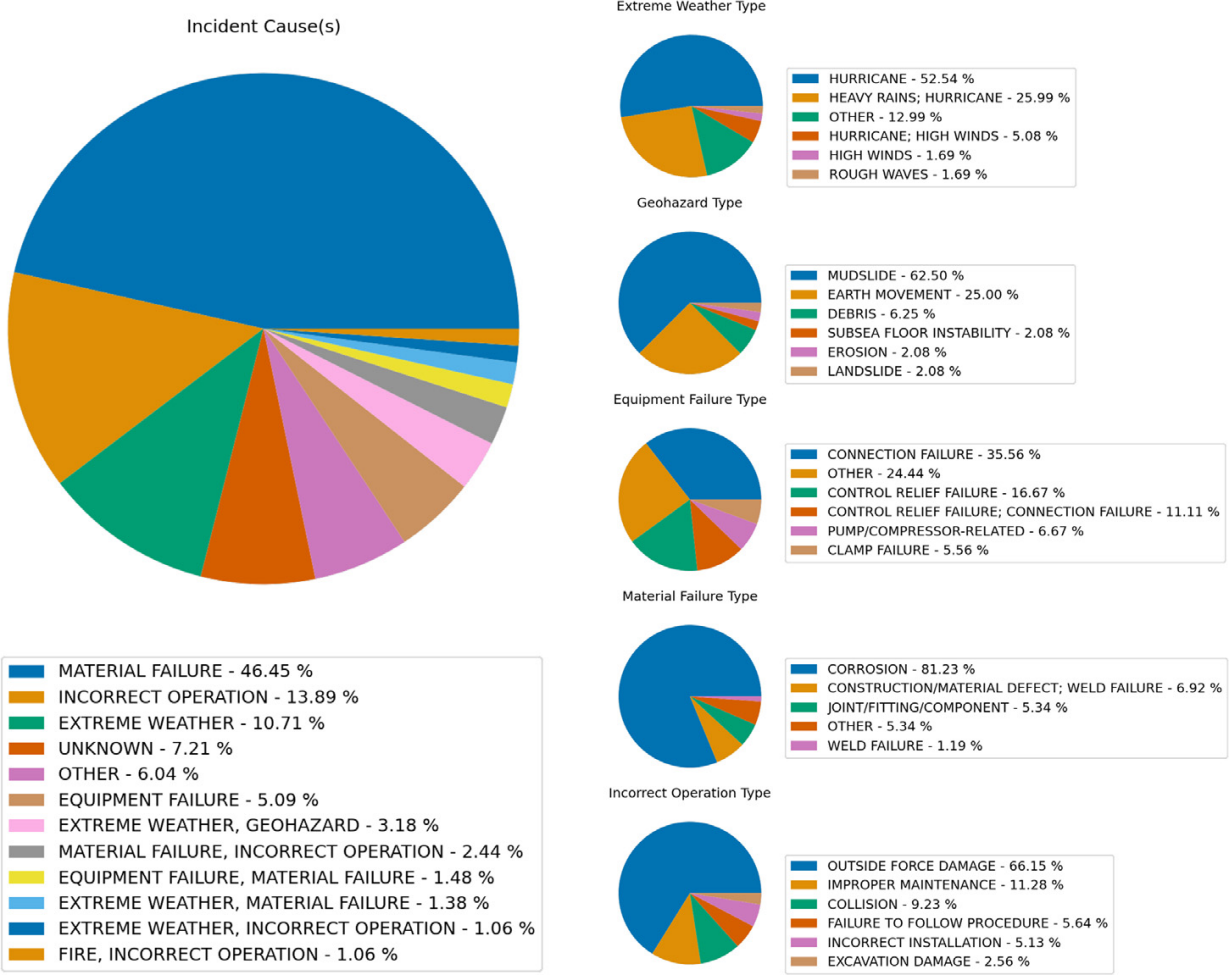
Pie charts representing potential incident cause and the potential incident sub-causes of extreme weather, geohazards, equipment failure, material failure, and incorrect operations.

The process of standardizing the disparate incident data required a manual evaluation of reported incident notes and comments to gather contextual information, which was then utilized to develop a protocol for aggregating potential events and factors into the existing potential cause fields. Following a thorough review of the reported incidents, keyword searches and manual curation were applied to determine which incidents were potentially caused by one or more of the following categories: extreme weather, geohazards, equipment failure, material failure, and incorrect operations. Variables that made up these categories, and their instance rate by report are shown in Fig. 4. By aggregating reported incident data, it is possible to examine the trends among potential incident causes. This allows for a better understanding of the sequence of events leading up to incidents. For example, the data suggests that material failure (e.g., corrosion) is the most common cause of reported incidents (Fig. 4). In addition, most of the reported incident records noted resulting in structural damage, corrective actions, or release events (Table 1).

### 3.4 Environmental loadings

Environmental loadings or metocean (meteorological and oceanographic) factors, such as wind, waves, currents, and biochemical quantities (e.g., dissolved inorganic nitrogen) impacts infrastructure integrity over time, as well as in short-term durations of extreme weather events (e.g., hurricanes). Studies have found long-term, cyclical loadings from currents and waves contribute to structural integrity loss including increased corrosion and crack propagation [5]. Material degradation, including corrosion, might also be caused by marine growth, which might increase with time [6]. Short-term extreme events have damaged, destabilized, and destroyed pipelines [7]. Likewise, joint metocean events such as deep topographic waves in conjunction with GoM Loop Current eddies have produced bottom-intensified ocean currents [8]. Corroborated with the incident reports, extreme weather can also act as a catalyst for cascading events resulting in ruptures, release events, and abandonment, including seafloor debris flows, and collisions with vessel anchors. For example, as a vessel anchors to weather a storm, it may get dragged due to currents and waves, causing the anchor to collide with a pipeline.

Accounting for the environmental load categories of wind, waves, currents (including ocean surface and seafloor bottom velocities), storms, and biochemical variables, a series of in situ measurements and model outputs were acquired from credible, publicly available resources. Variables for each environmental loading category and variable descriptions are provided in Table 2.

**Table 2:** Table of environmental loadings, variables, and variable definitions. Full citations of the associated resources are available in dataset metadata [3].

Environmental Loading	Variables	Description
Wind	u	East-west component of wind velocity (m/s) measured 10 m above water surface.
	V	North-south component of wind velocity (m/s) measured 10 m above water surface.
	Magnitude	Wind velocity magnitude (m/s) (i.e., speed) measured 10 m above water surface.
Waves	Height	Significant wave height (m) from combined winds and swell measured at the water surface.
	Period	Wave period (s) for wave height data.
	Direction	Wave direction (degrees true), as in 0°, greater than or equal to, coming from the north; 90°, greater than or equal to, coming from the east.
	Bottom Pressure	Pressure (kg/m^2^) at ocean bottom due to surface waves.
	Power	Wave energy flux (kW/m).
Ocean Surface Velocity	U	East-west ocean current velocity (m/s), measured at the water surface.
	V	North-south ocean current velocity (m/s), measured at the water surface.
	Magnitude	Ocean current velocity magnitude (m/s) (i.e., speed), measured at the water surface.
Ocean Bottom Velocity	U	East-west ocean current velocity (m/s), measured at ocean bottom.
	V	North-south ocean current velocity (m/s), measured at ocean bottom.
	Magnitude	Ocean current velocity magnitude (m/s) (i.e., speed), measured at ocean bottom.
Storms	Tropical Storms	Count of tropical storms, which spatially intersected infrastructure location during said structure's lifetime.
	Category 1	Count of Category 1 hurricanes (C1), which spatially intersected infrastructure location during said structure's lifetime.
	Category 2	Count of Category 2 hurricanes (C2), which spatially intersected infrastructure location during said structure's lifetime.
	Category 3	Count of Category 3 hurricanes (C3), which spatially intersected infrastructure location during said structure's lifetime.
	Category 4	Count of Category 4 hurricanes (C4), which spatially intersected infrastructure location during said structure's lifetime.
	Category 5	Count of Category 5 hurricanes (C5), which spatially intersected infrastructure location during said structure's lifetime.
	Maximum Sustained Wind Speed	Maximum sustained wind speed (knots) during spatially intersecting storm event.
	Maximum Reported Wind Gust	Maximum reported wind gust (knots) during spatially intersecting storm event.
	Minimum central Pressure	Minimum central pressure (mb) during spatially intersecting storm event.
	Hit Count	Total number of tropical storms and hurricanes that spatially intersected infrastructure's location during said structure's lifetime.
Biochemical	Alkalinity	Alkalinity (mEq/m^3^)
	Diatom Phytoplankton Chlorophyll	Chlorophyll concentration due to diatom phytoplankton (mg Chl/m^3^)
	Nondiatom Phytoplankton Chlorophyll	Chlorophyll concentration due to non-diatom phytoplankton (mg Chl/m^3^)
	Detritus	Detritus (mmol-N/m^3^)
	Dissolved Inorganic Carbon	Dissolved Inorganic Carbon (mmol-C/m^3^)
	Dissolved Inorganic Nitrogen	Dissolved Inorganic Nitrogen (mmol-N/m^3^)
	Detrital Carbon	Detrital Carbon (mmol-C/m^3^)
	Dissolved Iron	Dissolved Iron (mmol-Fe/m^3^)
	Dissolved Oxygen	Dissolved Oxygen (mmol-O2/m^3^)
	Biogenic Silicon	Biogenic Silicon (mmol-Si/m^3^)
	Diatom Phytoplankton	Diatom Phytoplankton (mmol-N/m^3^)
	Nondiatom Phytoplankton	Nondiatom Phytoplankton (mmol-N/m^3^)
	Silicate	Silicate (mmol-Si/m^3^)
	Meso Zooplankton	Meso Zooplankton (mmol-N/m^3^)
	Micro Zooplankton	Micro Zooplankton (mmol-N/m^3^)

Wind data were collected at a 2.5-degree resolution (approximately 260 km in the GoM) as daily and monthly aggregates for 1900 through 2010 from the National Center for Atmospheric Research European Center for Medium-Range Weather Forecasts Atmospheric Reanalysis of the 20th Century National Centers for Environmental Prediction. Variables of wind data include east-west and north-south wind velocity components 10 m above the water surface, from which a magnitude was computed.

Wave data were acquired through the National Oceanic and Atmospheric Administration's (NOAA) Wavewatch III Phase 2 and Production Hindcast resources. Wave variables including height, period, and direction were collected at a 0.5-degree spatial resolution as three-hour intervals for 1979 through 2019. Using these wave variables, wave bottom pressure was calculated for all pipeline locations.

Surface ocean velocity, including east-west and north-south components at the water surface were acquired from the Hybrid Coordinate Ocean Model (HyCOM) Gulf of Mexico. These data were collected at a 4 km spatial resolution at three-hour intervals for 2003 through 2019. In addition, bottom ocean velocity variables were also acquired from HyCOM Global with supplemental information from the Texas-Louisiana Continental Shelf Model (TXLA) with a horizontal resolution spanning from 0.65 km near the coast to 3.7 km near the outer continental slope. Bottom ocean velocity data were collected as daily instantaneous values for 1994 through 2015 from HyCOM Global, and from 2003 through 2021 from TXLA.

Meteorological storm data were acquired from the International Best Track Archive for Climate Stewardship's (IBTRACS) Tropical Cyclone Best Track Data. Storm-related variables from this resource included tropical storm and hurricane events and associated measurements including maximum reported wind gust, minimum central pressure, and maximum sustained windspeed. Storm data were acquired as a series of polyline tracks when storm events occurred from the beginning of the pipeline database to 2022.

Biochemical data, which are related to marine growth and corrosion were obtained from the Model of Ecosystem Dynamics, nutrient Utilization, Sequestration and Acidification (MEDUSA-2.0) from the National Oceanography Center in the United Kingdom. From MEDUSA-2.0, 15 variables were acquired including dissolved inorganic nitrogen, *meso* zooplankton, and diatom phytoplankton chlorophyll (Table 2). Biochemical measurements were acquired at a 10 km spatial resolution as 5-day aggregates for 1990 through 2015.

### 3.5 Seafloor factors & potential geohazards

Offshore pipelines are typically installed on the seabed or buried in sediment, making them potentially prone to seafloor instability and geohazards, such as subsea sediment flows or landslides. These events have caused damage and destruction to energy infrastructure, including pipelines and platforms, resulting in the unintended hydrocarbon release events. According to a study by Tian et al. events like hurricanes can disrupt sediments in shallow waters of the Gulf of Mexico, leading to seafloor instability and affecting pipeline placement [7]. Sediment characteristics such as thickness, bottom substrate, and historic landslide locations have an impact on sediment movement events and the potential for landslides [9]. As a result, these factors were incorporated into the pipeline locations dataset.

A sediment thickness spatial data layer entitled Sediment Thickness for North America and Neighboring Regions was obtained from the Central and Eastern U.S. Seismic Source Characterization for Nuclear Facilities Project Data Summary. This dataset provides sediment thickness in kilometers at 0.08-degree resolution (approximately 9.2 km).

Seafloor substrate data for the GoM were acquired from the usSEABED Integrated Sea-Floor-Characterization Database developed by the U.S. Geological Survey in cooperation with the Institute of Arctic and Alpine Research at the University of Colorado Boulder. Information obtained from this dataset includes sediment gravel, mud, and sand fraction content by weight and substrate rock presence as percent exposure as well as associated uncertainty measurements.

Historical landslide data, entitled Historic Submarine Landslides in the Northern Gulf of Mexico were acquired through the Energy Data eXchange® (https://edx.netl.doe.gov). This resource, developed by NETL, represents zones of depletion of past mass transport deposits for four GoM regions.

**Fig. 5:**
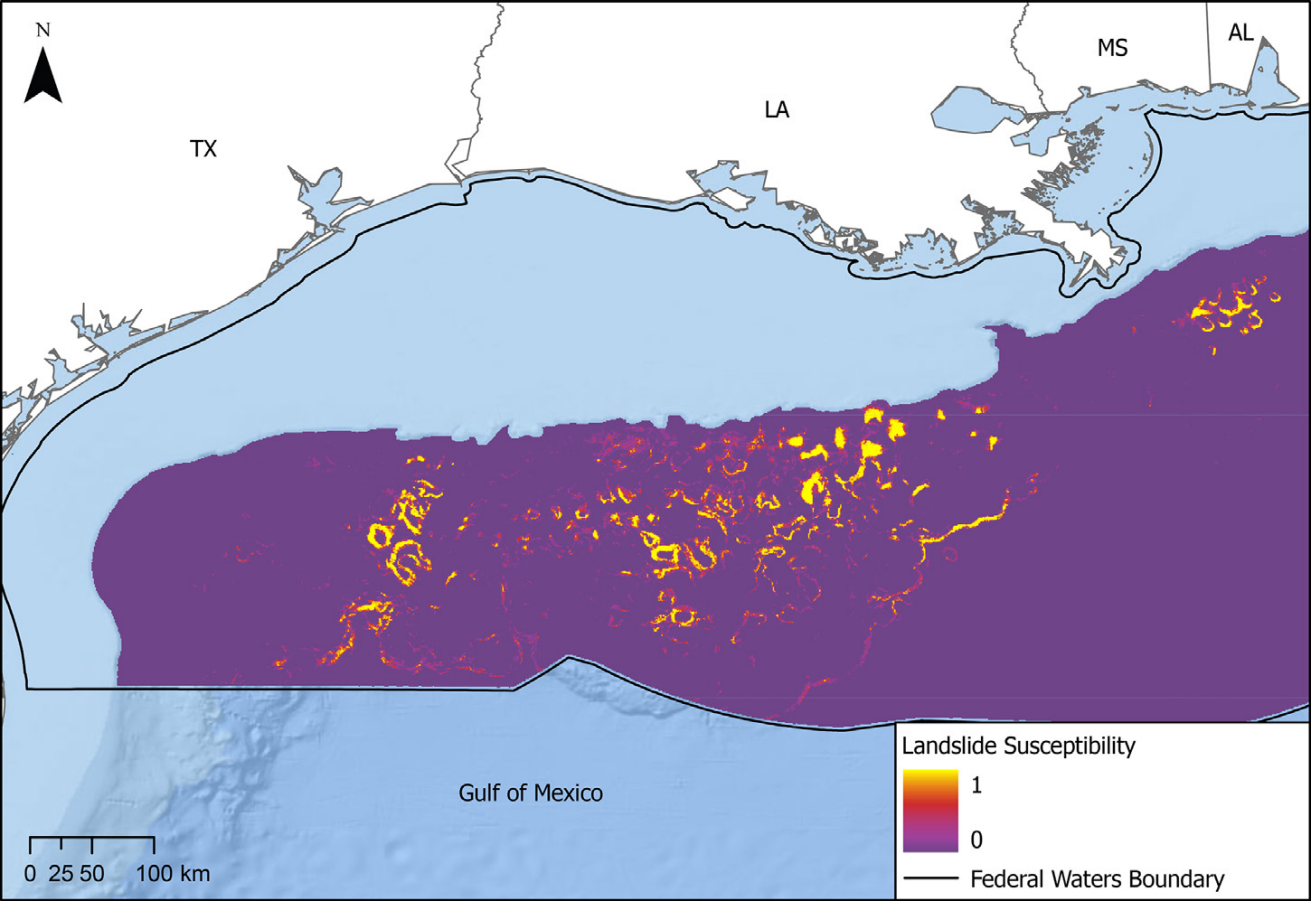
Map showing OGA landslide susceptibility results from the machine learning model within the GoM, with 0 representing least susceptible areas and 1 representing most susceptible areas.

Landslide susceptibility data were obtained from the landslide susceptibility analysis available through the Ocean & Geohazard Analysis tool (OGA) [10]. The OGA software is a data science-informed tool designed for metocean and seafloor hazard identification and risk assessment. Landslide susceptibility mapping was performed using a gradient-boosted decision tree algorithm to spatially model landslide probability over the U.S. Exclusive Economic Zone in the GoM where water depths exceed 120 m [9]. The machine learning model was trained on historical landslides, as well as topographical, geomorphological, geological, and geochemical factors to forecast where sediment instability is more likely to occur. As displayed in Fig. 5, the model outputs a raster surface with cell values ranging from 0 (less susceptible) to 1 (more susceptible), representing the predicted landslide susceptibility scores.

## 4 Experimental Design, Materials and Methods

Following the manual data collection from publicly available and credible resources, data were processed for dataset integration. This multistep processing included merging the disparate reported incident files, as well as preparing the resulting incident dataset, structural data, environmental loadings data, and the seafloor and geohazard data for integration. The original scope of the U.S. Gulf of Mexico Pipeline and Reported Incident Datasets, specifically the pipeline point locations dataset, was to train and test the multiple machine learning models of the Advanced Infrastructure Integrity Model to evaluate infrastructure integrity [11]. As these models require all variables representing various stressors to be in one dataset, this required the application of Geographic Information System (GIS) applications to process the data, and to spatially and, when applicable, temporally match the processed data to pipeline point locations.

**Fig. 6:**
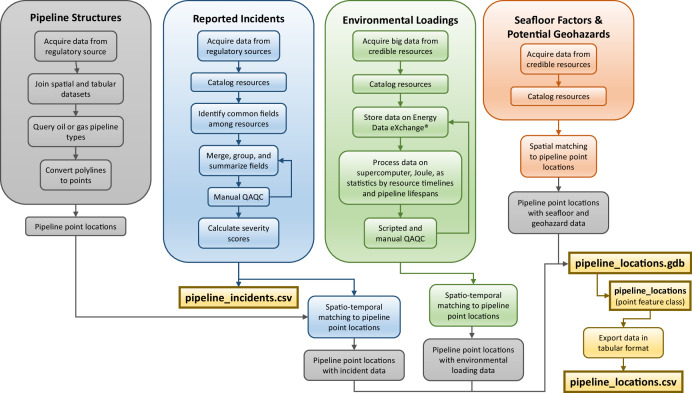
Workflow diagram illustrating data processing and integration methods, resulting in the published datasets.

Much of the processing was completed manually or through custom scripts, which were developed to fit the unique needs to each dataset. Processing, including quality assurance and quality control (QAQC), was completed using ESRI's ArcGIS Pro (version 3.1.3), Python (version 3), C++ (version C++14), Julia (version 1.8.2), and MATLAB (version R2023a). Processing using NETL's JOULE 2.0 Supercomputer (https://hpc.netl.doe.gov/) was required for the environmental loading data. The processing steps for the pipeline data, reported incidents, environmental loadings, and seafloor factors and potential geohazards are discussed in the following sections and the workflow is outlined in Fig. 6.

### 4.1 Pipeline structural data processing methods

Fig. 6 overviews the process for creating the pipeline point locations spatial and tabular datasets. Pipeline spatial data were obtained from the BSEE Data Center's Mapping webpage (https://www.data.bsee.gov/Main/Mapping.aspx) and consisted of a polyline feature class in a file geodatabase representing the geometry and geography of the pipelines. Tabular datasets that represented structural characteristics were obtained through the Pipeline Information webpage of the BSEE Data Center (https://www.data.bsee.gov/Main/Pipeline.aspx). Attributes from the tabular data were joined to the spatial data by the segment number, which is a unique number assigned to each pipeline segment for internal identification by the Minerals Management Service (MMS), which is the predecessor agency of BSEE. The pipeline data were filtered to only include pipelines with product code values that were oil- or gas-related. This resulted in a pipeline polyline dataset containing structural information, with polylines that ranged from less than a meter to more than 160 km in length.

Next, to accurately match all variables of interest (e.g., environmental loadings) across the length of a pipeline, a point layer was derived from the polyline feature class using GIS to create points along each pipeline per kilometer and at each pipeline end point. This resulted in 89,986 pipeline point locations, with each point containing all relevant data for the whole pipeline, with the ability to join data to explicit locations. Moreover, the points data can be mapped back to the pipeline polyline data using segment number.

Within the database, 61 % of the records contained installation dates and 44 % contained abandonment dates. With the original design purpose of the database to evaluate pipeline integrity as a function of remaining structural lifespan, installation and removal or abandonment date information were critical factors. Corroborated through annual reports and subsequent fields, it was noted that more pipelines were abandoned than the abandonment date field indicated. To fill the identified data gaps, proxy installation and abandonment dates were calculated. First, outliers were found and removed from the existing installation and abandonment dates using the interquartile range method with a scale of 1.5. Therefore, any dates that were found to be 1.5 times above the upper quartile range or 1.5 times below the lower quartile range were removed. To develop proxy installation dates, the average time between installation approval dates and installation dates were calculated from records where both values were available and was evaluated for correlation, resulting in a relatively strong correlation (R^2^ = 0.91) when comparing installation date and installation approval date values. Given this strong correlation, the new installation proxy field was filled first by the reported installation dates, where available, and then gaps were then filled based on installation approval date field values, where available. Next, pipeline records without abandonment date values, but containing status values of abandoned or removed were identified. To calculate abandonment proxy date, the average time between abandonment approval dates and abandonment dates were calculated from records where both values were available. This resulted in a relatively strong correlation (R^2^ = 0.88) between abandonment approval dates and abandonment dates. These processes resulted in 76 % of the installation proxy dates and 48 % of the abandonment proxy dates being filled.

### 4.2 Reported incident processing methods

Data from PHMSA are based on information acquired through report forms, which are required to be completed in the event of the release of hazardous materials from a pipeline. The incident report forms were released for various time periods between 1986 and 2021. The forms and resulting data are unique by the four reporting pipeline systems: Gas Distribution System Form PHMSA F 7100.1 (49 eCFR 191.9), Natural and Other Gas Transmission and Gathering Pipeline Systems Form PHMSA F 7100.2 (49 eCFR 191.15), Liquefied Natural Gas Facilities Form PHMSA F 7100.3 (49 eCFR 191.15), and Hazardous Liquid Pipeline Systems Form PHMSA F 7000-1 (49 eCFR 195.54). During an initial review of the reports, NETL found that the Gas Transmission and Gas Gathering (GTGG) and Hazardous Liquid (HL) reports included pipelines in the GoM, and therefore only data from these two pipeline systems were included in the reported incidents dataset.

The reporting structure differed with varying field names and definitions by pipeline systems and time periods. To consistently and accurately align the various PHMSA reports, fields across the systems and reporting time periods were catalogued to identify commonalities and map how incident data could be merged into one dataset. If two field names and definitions were identical, those fields were matched together. Otherwise, field definitions were compared through Python scripts utilizing open-source libraries such as TheFuzz, which measures similarity between two strings of text using Levenshtein Distance. Comparisons resulted in similarity scores ranging from 0 (no similarity) to 1 (exact match). For comparisons scoring greater than or equal to 0.5, manual curation was required for matching. Field pairs with scores less than 0.5 were disregarded. This process was first completed separately for each of the pipeline system types, then again applied to combine the GTGG and HL reports together. The resulting dataset contained 889 incident records and 455 fields.

Next, incident reports from BSEE were integrated. These data required significantly less processing, as over seven decades of incident data from BSEE and predecessor agencies (i.e., MMS and the Bureau of Ocean Energy Management, Regulation, and Enforcement) were previously processed and integrated for the published Comprehensive GOM Federal Waters Platform, Incident, Metocean, and Geohazard Dataset [12]. The already processed BSEE incident data were queried for pipeline-related incidents, resulting in 54 additional records for integration.

Fields among the PHMSA and BSEE data were manually matched including location, pipeline identifiers, commodity release type, and cause. Following initial compilation, fields were screened for redundancy and completeness. Fields containing only null values were removed and redundant fields were manually merged. Next, the dataset was cleaned to ensure consistency among data value, types, and units. For example, total cost fields were converted to 2021 USD to enable consistent cost comparison over time. This conversion followed PHMSA methods to convert costs using the Bureau of Economic Analysis, Government Printing Office (Chained) Price Index, Table 10.1 from Fiscal Year 2021 (https://www.govinfo.gov/app/collection/budget/2021).

Next, a QAQC protocol was established, recorded, and completed to validate the correct classification of incidents. This protocol included the manual review of reported narratives, notes, and cause descriptions, which were cross-examined against location, cost, commodity released, damage, potential cause, and remediation related fields. The contextual review enabled data gaps from the cross-examined fields to be filled and verified. Details on the fields included in the final datasets can be found in the associated field dictionaries [3]. In addition, during the QAQC, fields were added to the dataset to denote if the incident was platform-related, and if the incident led to pipeline abandonment.

Further limiting data gaps, some null values were filled by deriving data from similar fields where possible, and sparsely filled fields were combined into broad categorical fields. This included categorizing a subset of fields as potential causes (e.g., material failure, incorrect operation) or consequences (e.g., abandonment, structural damage) of the reported incident. The resulting potential cause and potential consequence fields were then utilized to calculate preliminary severity scores per incident.

Severity scores were added to the dataset to represent a standardized measurement of the reported economic, environmental, structural, and operational damage to the pipelines. Four severity scores were calculated. The first was based on reported incident cause and consequences, the second was based on reported consequences, the third was based on total cost (in 2021 USD), and the fourth was based on release amount. Preliminary weights were applied based on field values to calculate the first and second severity scores. As outlined in Table 3, the initial weights represent potential environmental, economic, and structural costs of the reported incident based on fields representing potential causes (e.g., release events) and consequences (e.g., release amount, fatalities, damage presence), which were limited by report completeness. Severity scores by reported total release amount and reported total cost were calculated by normalizing the value of interest across all incidents. For example, the incident with the highest cost received a severity score by reported total cost of 1, whereas an incident with the lowest reported cost was given a score of 0. Scoring methods were similar to those used to assess incidents related to offshore platforms in the GoM [13].

**Table 3:** Preliminary severity scores based on potential cause or consequence were based on a subset of the reported data fields. These severity scores were calculated per incident using an additive approach of summing all weights that apply. Next final scores across all incidents were normalized, resulting in ranges of 0 (low severity) to 1 (high severity). Field names with an asterisk (*) denote that if the pipeline was reported as destroyed (i.e., Pipeline Destroyed is marked as YES) the field value in question was ignored for scoring.

Field	Categorized as Potential Cause or Consequence	Field Value(s)	Applied Severity Weight
Structural Damage	Consequence	YES	1
Release Type*	Cause	Flare	1
		Leak	2
		Separated	4
		Severed	4
		Ruptured	4
		Destroyed	6
Fire	Consequence	YES	1
Explosion	Consequence	YES	2
Total Cost (2021 USD)	Consequence	<= $25,000	2
		> $25,000 & <= $1000,000	3
		> $1000,000 & <= $10,000,000	4
		> $10,000,000	5
Shutdown	Consequence	YES	1
Total Release Amount (Value ranges determined by 25 %, 50 %, 75 %, and 90 % quantiles)	Consequence	> 0 & <= 1.4	1
		> 1.4 & <= 67.4	2
		> 67.4 & <= 912.5	3
		> 912.5 & <= 4046.8	4
		> 4046.8	5
Injury Count	Consequence	> 0	1
Fatality Count	Consequence	> 0	2
Platform Damaged	Consequence	YES	2
Platform Destroyed *	Consequence	YES	4
Pipeline Destroyed	Consequence	YES	6
Abandonment *	Consequence	YES	6

Carrying over information found through the QAQC cross-examination, geographic fields including OCS lease block, latitude, and longitude, required additional processing. If location information (e.g., OCS lease block, coordinates, area code) was present in the narrative, notes, or description fields, the information was used to validate or fill the associated geographic fields. Next, a custom Python script was developed to clean the inconsistent latitude and longitude fields (e.g., converting degrees, minutes, and seconds to decimal degrees). Data were then mapped and spatial intersects were applied to infer the OCS lease block for each point, if not previously reported. This resulted in more than 90 % of the incident records containing filled OCS lease block information.

Incidents were spatially and temporally matched to pipeline points. Spatially, pipeline points were identified within the same lease block of an incident. Next the reported incident date was then checked against the pipeline install date and abandonment date, when available. If a reported incident occurred in the same lease block as the pipeline point and occurred after installation but before abandonment (if applicable), the incident was matched to said pipeline point. For all incidents that were matched to a pipeline location point, the incident data was summarized by calculating the total count of matched incidents for select binary fields (e.g., internal corrosion reported incident count by OCS lease block, hurricane reported incident count by OCS lease block), as well as sums of the four normalized severity scores. This method resulted in incidents occurring at one or more points and further analysis should be done at the OCS lease block-level due to spatial uncertainties at finer spatial resolutions.

### 4.3 Environmental loadings

Following data acquisition from open sources (Table 2), data were shared and stored in a private EDX workspace using the EDX application programming interface (API) [14]. Thousands of single network Common Data Form (NetCDF) files were aggregated to create a full-time series per dataset, then interpolated to each of the pipeline point locations, spanning the reported lifespan of each pipeline. Due to the large size of the datasets totalling nearly 400 GB, NETL's supercomputer, Joule, was used for the dataset processing. Each run utilized a single compute node consisting of 100 cores and approximately 192 GB of random-access memory (RAM). Some of the QAQC processes required a different computer with 512 GB of RAM.

Each dataset presented unique challenges such as the type of grid and the nature and number of missing values. Thus, the processing software, including the underlying logic was often adapted for each specific dataset. Heavy utilization of the C++ templating and namespace features aided in mitigating the differences between grid and data representations. Parallel processing of the data was achieved using the OpenMP framework. To smoothly interpolate over gaps in the data, a modified Akima spline was applied. To rapidly locate storm-infrastructure intersects, a quadtree with a depth of seven was implemented as a spatial sorting scheme.

Storm data from IBTRACS presents all available storm data for the GoM including storm trajectory, minimum central pressure, maximum sustained winds, and gust speeds among others. Due to the space-time dependence of storm trajectories, a storm visualizer was constructed to ensure that any given storm would intersect with the appropriate pipeline positions at the appropriate times. The visualizer validated that the logic for matching the correct storms to each pipeline location was correct.

Wind, wave, and current data were obtained from data assimilating models. Specifically, models which numerically solve the equations for motion in geophysical fluids were applied, including equations for thermodynamic variables, and all observations of the atmosphere and ocean were assimilated to ensure that the numerical solutions remain as close to reality as possible. Observations used for data assimilation include a variety of satellite and in situ datasets including Lagrangian observations (i.e., sensors that are freely moving with ocean currents and atmospheric winds).

Biochemical data were obtained from climatological in situ measurements from the World Ocean Atlas (WOA) 2018 [15] and from MEDUSA-2.0, a freely running ocean model with a biochemical component. The two biochemical datasets were compared, and MEDUSA-2.0 was selected for inclusion as it gave a good representation of the variables of interest and most importantly, the temporal and spatial coverage and resolution were considerably better than with the climatological WOA dataset.

Due to the nature of ocean models, the best representation of bottom velocity is obtained when the vertical coordinate is a terrain-following coordinate. HyCOM Global contains terrain-following coordinates near the bottom, and while the coverage is global, the resolution is somewhat coarse at 9 km in the GoM. The TXLA model also uses terrain-following coordinates (based on the Regional Ocean Modelling System) and has much higher resolution (horizontal resolution spans from 0.65 km near the coast to 3.7 km near the outer continental slope), but the TXLA model only covers the Louisiana-Texas shelf, approximately less than 500 m deep. Thus, TXLA was used where available and HyCOM Global in all other locations.

Bottom pressure induced by waves, expressed as a difference from hydrostatic pressure, was computed from maximum wave heights (not significant wave heights) as follows [16,17]

**Fig. 7:**
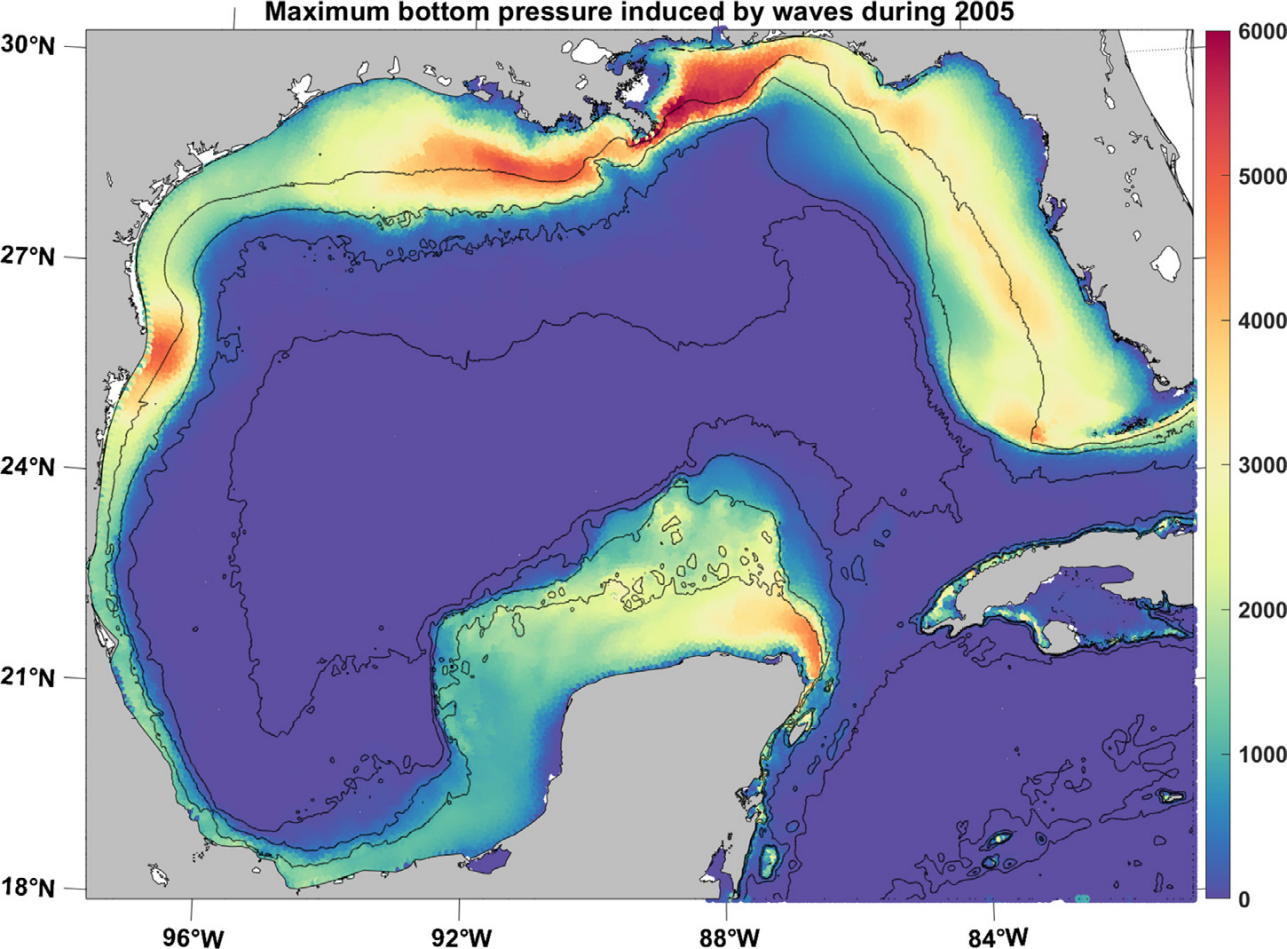
Maximum wave-induced bottom pressure (kg/m^2^) during 2005, as a function of longitude and latitude in the GoM. Black lines represent the 50-, 200-, 1000- and 3000-m isobaths.

P=0.67*ρ/2*Hcosh(2πD/L)

where *H* is the maximum wave height, *D* is the water depth, *L* is the wavelength, and ρ is a typical (constant) seawater density. As depth increases, the hyperbolic cosine goes to zero, resulting in bottom pressure becoming negligible around 200 m depth (Fig. 7).

After processing, data were checked for quality. This included creating a separate codebase in MATLAB, with some of the work done in Julia and in Python, ensuring that all computations were accurate, and that the data had been selected and processed correctly. The QAQC process was completed using a custom-built workstation with 512 GB RAM due to the size of the data and the incongruity among and within datasets. The resulting data includes metocean statistics specific to each pipeline point location. Statistics computed from the time series at each point included mean, median, standard deviation, maximum, minimum, and the 25th, 75th, 90th, and 99th percentiles. The total number of valid sample counts and the number of invalid records encountered were also tabulated and included.

### 4.4 Seafloor factors & potential geohazards data processing methods

Sediment thickness, seafloor substrate, historic landslide locations, and landslide susceptibility data were all acquired as spatial data with minimal data processing needed prior to integration into the pipeline point locations dataset. For inclusion, spatial reference systems of the original resources were spatially transformed into the geographic coordinate system, WGS 1984.

The seafloor and geohazard data layers were then spatially matched to the pipeline point locations in GIS. This resulted in key attributes (e.g., sediment thickness, seafloor substrate vales, landslide susceptibility) and data presence (e.g., presence of past submarine landslides) being spatially appended to the intersecting pipeline point locations.

### 4.5 Curation, metadata, and publishing

Following all processing, the datasets were curated for public release on EDX. The pipeline locations tabular dataset was converted into a file geodatabase with a point feature layer, and both CSV and file geodatabase were included for interoperability. Next, data dictionaries were created outlining field names, field definitions, field origins, units, and references where appropriate. Lastly, a QAQC of the data dictionaries was executed to ensure field names were consistent with the data, and definitions were reviewed for accuracy. A metadata document was also produced with details on the datasets including a description of the data, copywrite status, and points of contact. The datasets, field dictionaries, and metadata were then published on EDX and are publicly available to download.

## Limitations

As a compilation of existing resources, the accuracy of these datasets is limited by the original sources. For many of the resources obtained (e.g., structural data, reported incidents, environmental loadings), it should be noted that early reporting standards and methods have a greater uncertainty and are typically less complete or accurate when compared to more recent records. Moreover, the integration of data from disparate resources resulted in notable data gaps throughout the datasets. In addition, incident data from regulatory agencies rely on the accurate reporting of the incidents. For example, reported latitude and longitude fields were found to be highly uncertain and often outside of the reported lease block. Therefore, OCS lease block was used to spatially match reported incidents to pipeline locations. For the environmental loadings data, there are inherent assumptions from using interpolation methods to fill data gaps. Additionally, spatial resolution limits precision when matching environmental loadings to pipeline locations.

## Ethics Statement

The authors have read and follow the ethical requirements for publication in Data in Brief and confirming that the current work does not involve human subjects, animal experiments, or any data collected from social media platforms.

## CRediT authorship contribution statement

**Isabelle Pfander:** Methodology, Software, Validation, Investigation, Data curation, Writing – original draft, Visualization. **Lucy Romeo:** Conceptualization, Methodology, Software, Validation, Investigation, Data curation, Writing – original draft, Visualization, Supervision, Project administration, Funding acquisition. **Rodrigo Duran:** Methodology, Software, Validation, Investigation, Data curation, Writing – original draft, Writing – review & editing. **Alec Dyer:** Methodology, Software, Validation, Writing – original draft, Writing – review & editing. **Catherine Schooley:** Data curation, Validation, Writing – original draft, Writing – review & editing. **Madison Wenzlick:** Methodology, Validation, Writing – review & editing. **Patrick Wingo:** Methodology, Software, Validation, Writing – original draft, Writing – review & editing. **Dakota Zaengle:** Software, Validation, Writing – original draft, Writing – review & editing. **Jennifer Bauer:** Conceptualization, Writing – review & editing, Supervision, Project administration, Funding acquisition.

## Data Availability

U.S. Gulf of Mexico Pipeline and Reported Incident Datasets (Original data) (Energy Data Exchange (EDX))
